# Supplementary material for: Large scale genomic rearrangements in selected Arabidopsis thaliana T-DNA lines are caused by T-DNA insertion mutagenesis
Source: BMC Genomics. 2021 Aug 6;22:599. doi: 10.1186/s12864-021-07877-8 (PMC8348815; doi:10.1186/s12864-021-07877-8)

Additional file 6: Structure of genomic locus around one insertion in GK-909H04.

Black lines indicate the TAIR9 and Col-0_GKat-wt genome sequences, red block represents the T-DNA, orange arrows indicate the positions of mapped ONT reads, blue blocks indicate the position of repeats and contain their length in bp.

locus 909H04-At1g38212

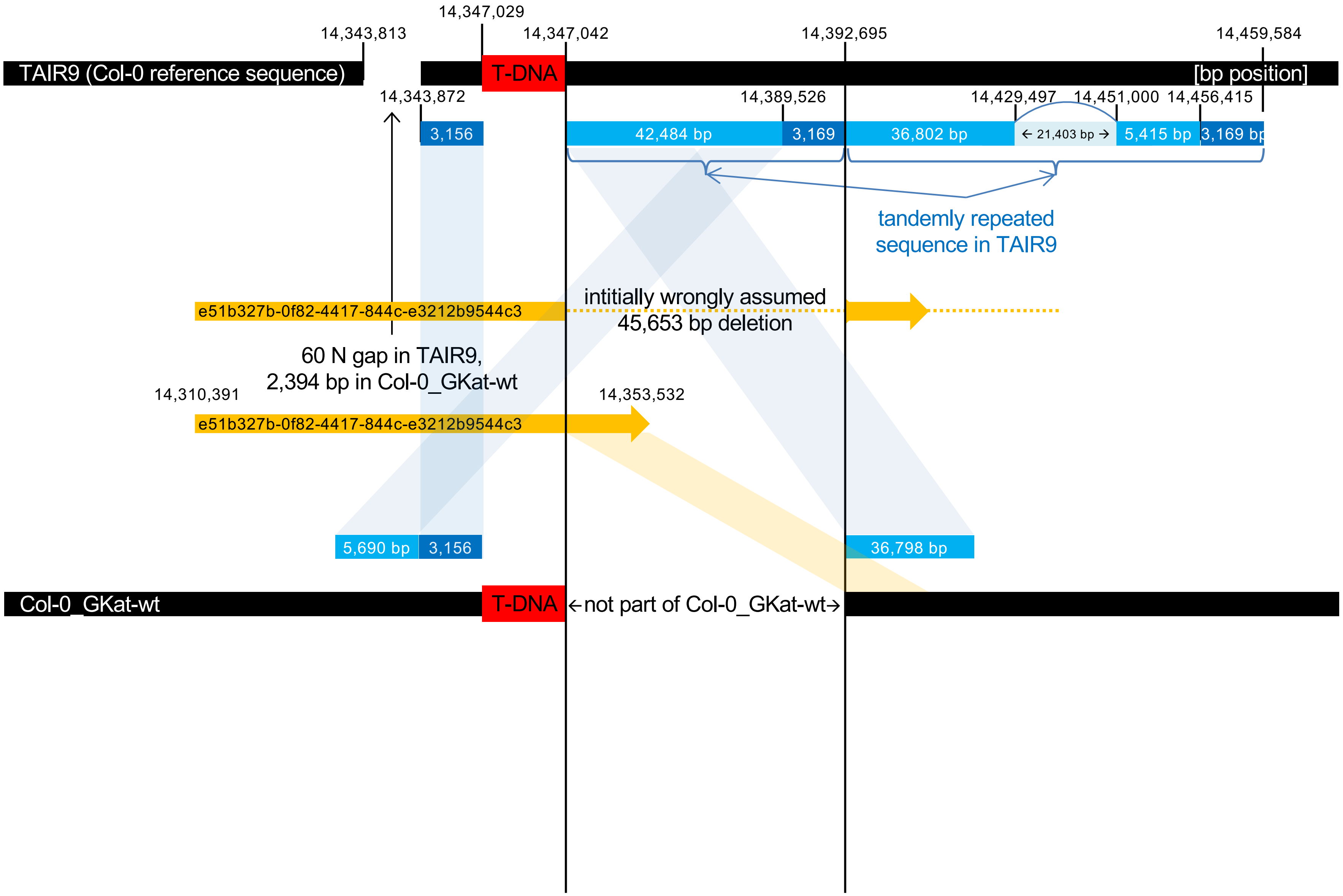

Supplement: Supplementary file 6 — Additional file 6. Structure of genomic locus around one insertion in GK-909H04. [file 12864_2021_7877_MOESM6_ESM.pdf]
