# Supplementary material for: Large scale genomic rearrangements in selected Arabidopsis thaliana T-DNA lines are caused by T-DNA insertion mutagenesis
Source: BMC Genomics. 2021 Aug 6;22:599. doi: 10.1186/s12864-021-07877-8 (PMC8348815; doi:10.1186/s12864-021-07877-8)
Supplement: Supplementary file 9 — Additional file 9. Assembly statistics of Col-0_GK-wt. [file 12864_2021_7877_MOESM9_ESM.docx]

**Additional file 9: Assembly statistics of Col-0_GK-wt**

**Assembly statistics of the Col-0_GKat-wt assembly (GCA_905067165)**

number of contigs: 35

average contig length [bp]: 3,545,375

minimal contig length [bp]: 106,611

maximal contig length [bp]: 16,157,556

total number of bases: 124,088,133

GC content [%]: 36.43

N25 [bp]: 15,112,119

N50 [bp]: 14,300,866

N75 [bp]: 11,323,513

N90 [bp]: 4,269,132
